# Efficacy and safety of temozolomide-based regimens in advanced pancreatic neuroendocrine tumors: a systematic review and meta-analysis

**DOI:** 10.1186/s12885-024-11926-2

**Published:** 2024-02-12

**Authors:** Erfan Taherifard, Muhammad Bakhtiar, Mahnoor Mahnoor, Rabeea Ahmed, Ludimila Cavalcante, Janie Zhang, Anwaar Saeed

**Affiliations:** 1 Department of Medicine, Division of Hematology & Oncology, University of Pittsburgh Medical Center, Pittsburgh, PA USA; 2 Novant Health Cancer Institute, Charlotte, NC USA; 3 UPMC Hillman Cancer Center, Pittsburgh, PA USA

**Keywords:** Neuroendocrine tumors, Pancreatic neoplasms, Temozolomide, Response evaluation criteria in solid tumors, Drug-related side effects, Adverse reactions

## Abstract

**Background:**

Recent advances in the management of pancreatic neuroendocrine tumors (pNETs) highlight the potential benefits of temozolomide, an alkylating agent, for these patients. In this meta-analysis, we aimed to assess the outcome of temozolomide, alone or in combination with other anticancer medications in patients with advanced pNET.

**Methods:**

Online databases of PubMed, Web of Science, Embase, the Cochrane Library, and ClinicalTrials.gov were searched systematically for clinical trials that reported the efficacy and safety of temozolomide in patients with advanced pNET. Random-effect model was utilized to estimate pooled rates of outcomes based on Response Evaluation Criteria in Solid Tumors criteria, biochemical response, and adverse events (AEs).

**Results:**

A total of 14 studies, providing details of 441 individuals with advanced pNET, were included. The quantitative analyses showed a pooled objective response rate (ORR) of 41.2% (95% confidence interval, CI, of 32.4%-50.6%), disease control rate (DCR) of 85.3% (95% CI of 74.9%-91.9%), and a more than 50% decrease from baseline chromogranin A levels of 44.9% (95% CI of 31.6%-49.0%). Regarding safety, the results showed that the pooled rates of nonserious AEs and serious AEs were 93.8% (95% CI of 88.3%-96.8%) and 23.7% (95% CI of 12.0%-41.5%), respectively. The main severe AEs encompassed hematological toxicities.

**Conclusions:**

In conclusion, our meta-analysis suggests that treatment with temozolomide, either as a monotherapy or in combination with other anticancer treatments might be an effective and relatively safe option for patients with advanced locally unresectable and metastatic pNET. However, additional clinical trials are required to further strengthen these findings. This study has been registered in PROSPERO (CRD42023409280).

**Graphical Abstract:**

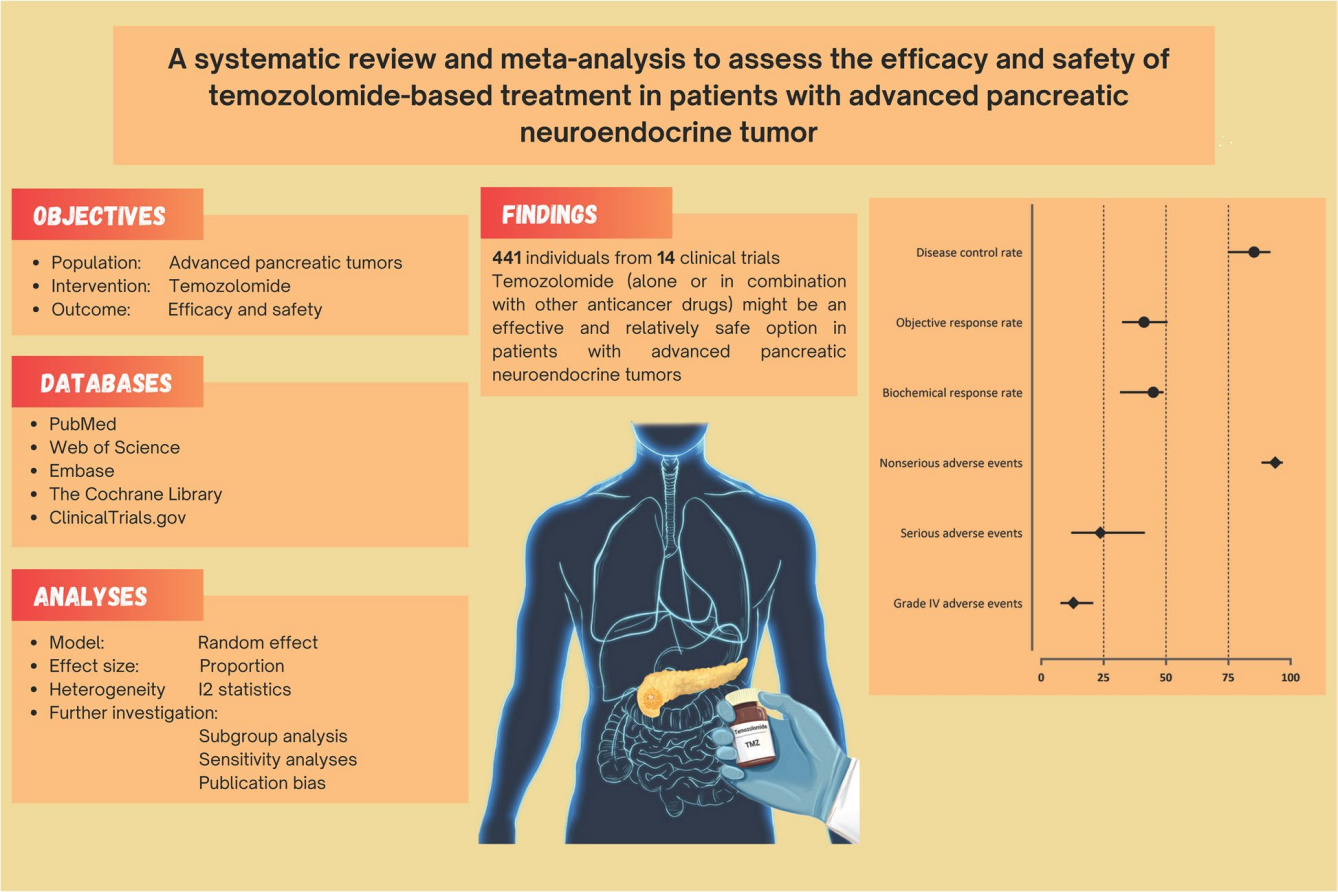

**Supplementary Information:**

The online version contains supplementary material available at 10.1186/s12885-024-11926-2.

## Introduction

Pancreatic neuroendocrine tumors (pNETs) are comprised of a heterogeneous group of tumors arising from multipotent neuroendocrine cells within the pancreatic islets. While these tumors represent a small fraction, less than two percent, of all pancreatic tumors, the incidence of pNETs has shown a substantial increase, with age-adjusted rates increasing nearly five-fold over the recent decades [1, 2]. pNETs exhibit diverse biological behaviors, ranging from indolent lesions to aggressive and poorly differentiated neoplasms [3]. Furthermore, these tumors can be either functional or nonfunctional, which greatly influences their clinical characteristics. Functional pNETs are characterized by the secretion of hormones such as insulin, glucagon, or somatostatin, leading to distinct clinical syndromes [4]. In contrast, nonfunctional pNETs, which constitute the majority of pNETs, typically do not present with hormone-related symptoms. Thus, early detection is more challenging, and these tumors tend to present at later stages [5].

Therefore, pNETs, although relatively rare, could pose a significant clinical challenge in both the diagnosis and the treatment [6]. Patients with pNET typically have a more favorable outcome compared to individuals with the more common type of pancreatic tumor, pancreatic adenocarcinoma [4]. The treatment and management of pNETs involve a variety of different therapeutic options, including surgical and medical approaches [7, 8]. Surgical intervention is the cornerstone of the management of these patients. It holds a potential for cure in patients with localized pNET. Additionally, surgical interventions could be beneficial in achieving significant symptom control, preserving function in metastatic organs, and ultimately impacting overall survival in patients with metastatic lesions [9–11]. In these patients, medical treatments play a crucial role in reducing the morbidity and mortality associated with pNETs [8]. These include the use of cytotoxic chemotherapy, somatostatin analogs, targeted therapies including monoclonal antibodies and small molecule inhibitors, and peptide receptor radionuclide therapy (PRRT) [7].

In recent years, there has been a growing body of evidence on the efficacy and safety profile of the use of temozolomide in patients with pNET. Numerous case series, observational studies, randomized (RCTs), and nonrandomized clinical trials with different levels of evidence suggest that temozolomide may result in favorable responses either alone or in combination with other medical therapeutic options in patients with advanced pNET [12, 13]. Temozolomide is an oral anticancer medication in the class of alkylating agents that was primarily used in patients with glioblastoma [14, 15]. This medication acts through methylation of deoxyribonucleic acid (DNA), particularly at the guanine residues, resulting in base pair mismatch, single- and double-strand break of the DNA, and eventually activation of programmed cell death [16]. However, unlike other alkylating agents such as streptozocin and dacarbazine used in these patients, temozolomide causes less cumulative toxicity such as myelotoxicity [12, 17, 18]. In this comprehensive systematic review and meta-analysis, we aimed to investigate the efficacy and safety of temozolomide for patients with advanced pNET. To assess efficacy, we have adopted the Response Evaluation Criteria in Solid Tumors (RECIST) [19]. Given the limited therapeutic alternatives often available to these patients, our study may shed light on the potential benefits of this treatment option and could guide physicians to enhance patient care in the future.

## Methods

This study was reported using the updated guideline of Preferred Reporting Items for Systematic Review and Meta-Analysis (PRISMA) [20]. The protocol for this study has been registered in PROSPERO [CRD42023409280].

### Systematic search

In this systematic review and meta-analysis, we selected MEDLINE (via PubMed), Embase, the Cochrane Library, Web of Science, and ClinicalTrials.gov databases for the literature search. To develop the search strategy, we built two groups of terms that were related to pNET and temozolomide. The group of terms related to pNET consisted of “Pancreatic neuroendocrine tumor”, “Pancreatic neoplasm”, “PNET”, “Pancreatic NET”, “islet cell carcinoma”, “islet cell tumor”, “Gastrinoma”, “Insulinoma”, “Glucagonoma”, “VIPoma”, and “Somatostatinoma”. The terms that were considered for temozolomide were “Temozolomide”, “Temodar”, “TMZ”, “Methazolastone”, “Temodal”, “CCRG 81045”, “NSC 362856”, “M and B 39831”, and “M & B 39831”. A combination of these terms was searched using Boolean operators of “AND” and “OR” and wildcard operators of “*/#” in the title, abstract, and keywords in the databases. We restricted the search results to clinical trial articles and articles in the English language to keep the search relevant. The full search strategy in each database is provided in the Supplementary material.

The search was initially conducted on March 2023 and then was updated on 29th of September, 2023. The reference list of the relevant records and review articles was also checked manually for articles in line with the objectives of our systematic review and meta-analysis.

### Eligibility criteria

Eligible papers for this review study were original trial studies, either RCTs or nonrandomized clinical trials. The trials should have been on human participants with an established diagnosis of locally unresectable or metastatic pNET. No restrictions were imposed based on the grade, functionality, or subtype of the pNETs. We included the trials that evaluated the effect of temozolomide, used either as monotherapy or in combination with other anticancer medications, in those with pNET. We only included papers in which their outcome measures related to the efficacy of treatment were assessed according to the RECIST criteria. Commentaries, letters to the editor, and correspondences were excluded unless they provided original data. We did not exclude any article based on the age group of the participants, their gender, country, etc. However, we only included articles that were written in English.

### Screening and data extraction

All the records were uploaded to Rayyan.ai. Duplicates were detected by the built-in Rayyan tool. After removing the duplicates, each record was reviewed using the title and abstract to exclude the unrelated record. Then, the full text of the studies left behind was read to assess whether they fulfilled the eligibility criteria. The process of screening was conducted by two reviewers (M.B. and M.M.), independently. Disagreements between the reviewers were resolved by discussion and consultation with the corresponding author. Once the studies were finalized for the systematic review and meta-analyses, 2 reviewers (M.B. and M.M.) independently extracted data from the eligible studies and entered it into an Excel spreadsheet. The corresponding author was consulted in the case of any inconsistencies or uncertainties regarding the screening and data extraction process.

The following data were extracted from the eligible studies: the title of the article, year of publication, design of the study, arms of the study and their characteristics, number of the participants in each arm, age and gender of the participants, information regarding prior chemotherapies, specifics of drug combinations, the dosage and duration of drug administration, and the outcome measures. The outcome measures we sought to extract from the articles in this study consisted of outcomes related to efficacy and safety. Efficacy-related outcomes included complete response (CR), partial response (PR), stable disease (SD), progressive disease (PD), objective response rate (ORR), disease control rate (DCR), and progression-free survival (PFS), overall survival and biochemical response, having more than 50% decrease from baseline chromogranin A levels. Safety-related outcomes encompassed the occurrence of any adverse events (AEs), as well as the specific type, rate, and grading of these events (grade 1 to 5), which were assessed and reported according to the National Cancer Institute Common Terminology Criteria for Adverse Events [21].

### Risk of bias assessment

In this systematic review and meta-analysis, we conducted a rigorous quality assessment of the included studies using assessment tools developed by the National Heart, Lung, and Blood Institute [22]. Specifically, we utilized the ‘Quality Assessment Tool for Before-After (Pre-Post) Studies with No Control Group’ and the ‘Quality Assessment of Controlled Intervention Studies’. Using these tools, different aspects of a study, including its design, statistical power, research conduct, data analysis, accuracy, and reporting are assessed. M.B., M.M., and R.A. performed the quality assessment, independently.

### Data synthesis

We conducted data management and statistical analyses using Comprehensive Meta-Analysis (Biostat Inc., CO, USA) version 3 and R statistical software version 4.3.1 (R Core Team, Austria). A random-effect-model was employed to estimate pooled effect sizes for various outcomes, including CR, PR, SD, PD, ORR, DCR, having more than 50% decrease from baseline chromogranin A levels, the incidence of nonserious AEs, the incidence of serious AEs, the incidence of grade 4 AEs, and the incidence of each specific AE. Grade 1 and grade 2 were considered as nonserious AEs, and grade 3 and grade 4 were categorized as serious AEs. We presented individual and pooled effect sizes in forest plots and included relevant statistics for each study, such as its 95% confidence intervals (CIs) and relative weight. To enhance clarity and ease of interpretation, the results of quantitative analyses for AEs were shown through a single plot. The visualization of this single plot was implemented using ggplot2 and dplyr libraries in R. Heterogeneity was assessed using the I^2^ statistic. Subgroup analysis was performed for ORR and DCR outcomes, focusing on the specific combination treatments received by patients. This allowed us to present these outcomes separately for patients who received temozolomide and bevacizumab combination-based treatment and those who received temozolomide and capecitabine combination-based treatment. We also conducted sensitivity analyses using the ‘leave-one-out’ method and assessed publication bias using Egger’s test.

## Results

### Study selection

**Fig. 1:**
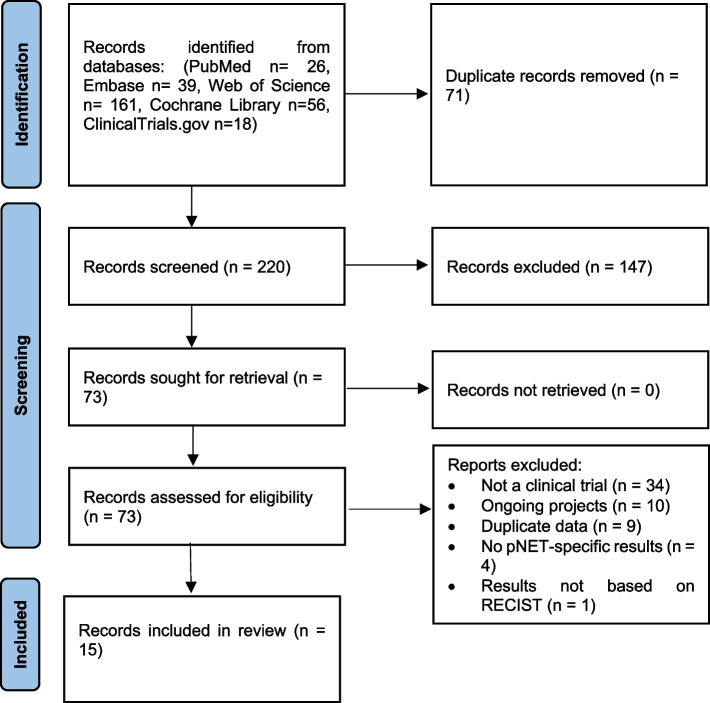
PRISMA flowchart of study identification, screening, and selection process

A total of 291 records were imported to our library, with 26 obtained from PubMed, 39 from Embase, 161 from Web of Science, 56 from the Cochrane Library, and 18 from ClinicalTrials.gov. After an initial screening process, which involved removing duplicate and irrelevant records based on their titles and abstracts, 73 records remained for a secondary screening based on their full text. Of these, only 15 containing details of 14 studies met the eligibility criteria for the quantitative analyses [23–37]. The remaining 38 records were excluded for various reasons: 32 were retrospective studies, 2 were prospective observational studies, 10 were ongoing research projects related to the use of temozolomide in combination with other anticancer medications in patients with neuroendocrine tumors including pNET, 9 presented data from the same population as the included studies, 4 did not report results for patients with pNET separately and the response to treatment was provided alongside other tumors, and one study did not provide outcomes based on RECIST criteria (Fig. 1).

### Study characteristics

The 15 included records corresponded to 14 studies, with two of the included records referring to a single study [34, 35]. We acquired data from these 14 studies for our systematic review and meta-analyses, primarily from the published articles. Additionally, information from ClinicalTrial.gov website was utilized for studies by Fine et al. (NCT00869050) [27], Pavel et al. (NCT02231762) [29], Bhave et al. (NCT01465659) [31], Shaheen et al. (NCT01525082) [33], and Pavlakis et al. (NCT02358356) [34, 35]. These studies collectively had information on a total of 570 patients with various advanced neuroendocrine tumors. Among these patients, 441 individuals had advanced locally unresectable or metastatic pNET, and radiologic and biochemical responses were reported for 414 and 49 of them, respectively. The majority of the included studies, 57.1%, were from the United States of America. The reports were published between 2006 and 2023. All of the studies were on the adult population, with median ages predominantly falling within the 50s and 60s (Table 1).

**Table 1:** Characteristics of the included studies assessing the response to temozolomide-based chemotherapeutic regimens in patients with pNET

Study, year and country	Study design	Characteristics of patients with pNET	Treatment	Number of evaluable patients	Age Median (range) Mean (± SD)	Male (%)	Patients who received prior chemotherapy (%)	PFS (median months, 95% CI)
Kulke et al. (a) 2006, US [23]	Single arm trial	Patients with locally unresectable or metastatic pNET	TMZ (150mg/m2 for 7 days every other week), and thalidomide (50-400mg daily)	11	56 (28-78)^a^	62^a^	45^a^	NE
Kulke et al. (b) 2006, US [24]	Single arm trial	Patients with advanced pNET	TMZ (150 mg/m2 for 7 days), and bevacizumab (5 mg/kg) every other week	17	61 (37-75)^a^	56^a^	35^a^	Not reported
Chan et al. (a) 2012, US [25]	Single arm trial	Patients with locally unresectable or metastatic pNET	TMZ (150mg/m2 for 7 days), and bevacizumab (5mg/kg on days 1) every other week	15	60 (36-74)^a^	56^a^	21^a^	14.3 (8.5- NE)
Chan et al. (b) 2013, US [26]	Single arm trial	Patients with low or intermediate grade locally unresectable or metastatic pNET	TMZ (150mg/m2 for 7 days every other week), and everolimus (5-10m per day)	40	53 (28-87)	60	23	15.4 (9.4-20.4)
Fine et al. 2014, US [27] NCT00869050	Single arm trial	Patients with progressive, differentiated, metastatic pNET	TMZ (150-200mg/m2 on days 10-14), and capecitabine (1500/m2 on days 1-14) each 4 weeks	11	30-89	39^a^	Not reported	> 18.2
Claringbold et al. 2016, Australia [28]	Single arm trial	Patients with low or intermediate grade locally unresectable or metastatic pNET	Capecitabine (1500mg/m2 for 14 days), TMZ (200mg/m2 in the last 5 days of capecitabine) both commenced 5 days before 177Lu-octreotate each 8 weeks	30	60 (38-78)	60	13	48
Pavel et al. 2018, Germany [29] NCT02231762	Single arm trial	Patients with low or intermediate grade unresectable, progressive pNET	TMZ (150 mg/m2 for 5 days in the first month, and 200 mg/m2 in the next months), and lanreotide Autogel (120mg) each 4 weeks	16	63.1 (± 11.0)^a^	58^a^	Not reported	11.1 (8.3-NE)
Cheng et al. 2018, China [30]	Parallel non-randomized trial	Patients with well-differentiated locally advanced or metastatic pNET	TMZ (150-200mg/m2 on days 1-7), and Endostar (15mg on days 1-14) each 3 weeks	6	48 (37–71)	57	7	12 (0.0-30.1)
			Dacarbazine (250mg on days 1-5), 5-FU (500mg on days 1-5), and Endostar (15mg on days 1-14) each 3 weeks	8				
Bhave et al. 2018, US [31] NCT01465659	Single arm trial	Patients with locally unresectable well differentiated pNET	TMZ (75-150mg/m2 for 7 days every other week), and pazopanib (400mg daily)	26	Above 18	52	Not reported	12.1 (5.7–25.1)
Kobayashi et al. 2021, Japan [32]	Single arm trial	Patients with locally unresectable or metastatic pNET	TMZ (200 mg/m2 on days 1-5) each 4 weeks	3	65 (40-75)^a^	46^a^	100	2
Shaheen et al., US NCT01525082 [33]	Single arm trial	Patients with low or intermediate grade locally unresectable or metastatic pNET	TMZ (on days 10-14), capecitabine (on days 1-14), and bevacizumab (on days 1 and 15) each 4 weeks	19	53.9 (± 10.7)	55	Not reported	Mean: 25.2 (18.7- 31.7)
Pavlakis et al. Austria [34, 35] NCT02358356	Randomized clinical trial	Patients with low or intermediate grade locally unresectable or metastatic pNET	TMZ (150mg/m2 on days 10-14), and capecitabine (1500mg/m2 on days 10-14) each 4 weeks	9	NR	NR	Not reported	Not reported
			TMZ (150mg/m2 on days 10-14), and capecitabine (1500mg/m2 on days 10-14), and 177Lu-octreotate on day 10 each 8 weeks	19				
Chi et al. 2022, China [36]	Randomized clinical trial	Patients with low or intermediate grade locally unresectable or metastatic pNET	TMZ (200mg on days 10-14), and tegafur (80-120mg on days 1-14) each 3 weeks	25	50 (45- 59.5)	63	7	16.2 (7.2- NE)
			TMZ (200mg on days 10-14), tegafur (80-120mg on days 1-14), and thalidomide (100mg on days 1-7, 200mg on day 8-14 and 300mg from day 15) each 3 weeks	27	50.5 (43.5-57.2)	60	10	NE (7.1- EN)
Kunz et al. 2023, US [37]	Randomized clinical trial	Patients with low or intermediate grade locally unresectable or metastatic pNET	TMZ (200mg/m2 on days 1-5) each 4 weeks	65	Median: 60.3 (± 6 11.5)	54	Not reported	14.4
			TMZ (200mg/m2 on days 1-5), and capecitabine (1500mg/m2 on days 10-14) each 4 weeks	68	Median: 61 (± 10.9)	56		22.7

*CI* confidence interval;*5-FU* 5-fluorouracil, *NE* not evaluable, *PFS* progression-free survival, *pNET* pancreatic neuroendocrine tumor, *SD* standard deviation, *TMZ* temozolomide, *US* United States

^a^ indicates that the value belongs to the entire study population, including individuals with pNET and individuals with other types of tumors based on the study

### Quantitative analyses on the efficacy of temozolomide combination therapy

**Fig. 2:**
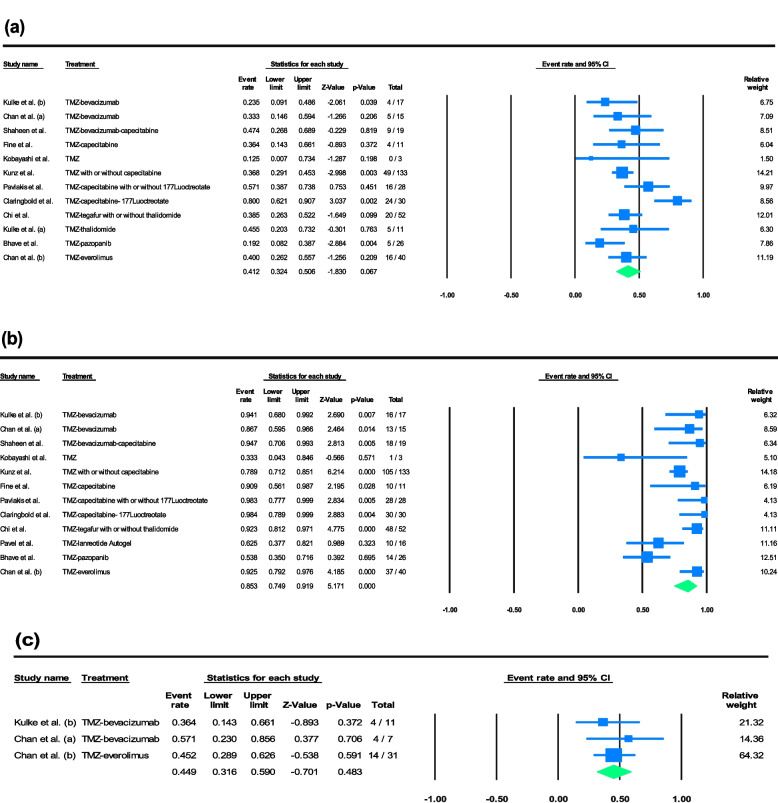
Forest plots of individual and pooled effect sizes of **a** ORR, **b** DCR, and **c** having more than 50% decrease in chromogranin A levels

Figure 2 demonstrates the forest plots for the pooled estimate of ORR, DCR, and biochemical response of having more than a 50% decrease from baseline chromogranin A levels. The analyses showed a pooled ORR of 41.2% (95% CI of 32.4% to 50.6%, I^2^ = 59.7%), a pooled DCR of 85.3% (95% CI of 74.9% to 91.9%, I^2^ = 69.3%), and a biochemical response of 44.9% (95% CI of 31.6% to 49.0%, I^2^ = 0.00%) (Fig. 2a-c). We also estimated the pooled rates of CR (4.7% with 95% CI of 2.5% to 8.9%, I^2^ = 14.4%), PR (37.9% with 95% CI of 30.6% to 45.8%, I^2^ = 39.9%), SD (45.4% with 95% CI of 38.2% to 52.9%, I^2^ = 38.7%), and PD (11.8% with 95% CI of 6.7% to 20.0%, I^2^ = 52.7%).

**Fig. 3:**
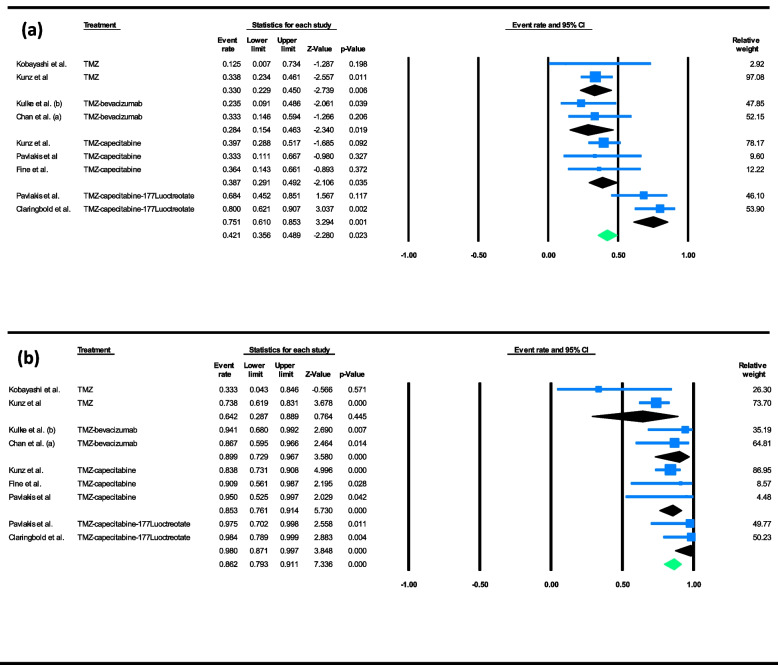
Forest plots of pooled rates for **a** ORR, and **b** DCR within subgroups for combination-based treatment administrated to the participants

Subgroup analyses were conducted for outcome measures of ORR and DCR based on the chemotherapy combination-based treatment assigned to the patients. Figure 3 displays the forest plots of these analyses for ORR and DCR. Temozolomide alone was associated with an ORR of 33.0% (95% CI of 22.9% to 45.0%, I^2^ = 0.00%) and a DCR of 64.2% (95% CI of 28.7% to 88.9%, I^2^ = 47.29%), temozolomide and bevacizumab with 28.4% (95% CI of 15.4% to 46.3%, I^2^ = 0.00%) and 89.9% (95% CI of 72.9% to 96.7%, I^2^ = 0.00%), temozolomide and capecitabine with 38.7% (95% CI of 29.1% to 49.2%, I^2^ = 0.00%) and 85.3% (95% CI of 76.1% to 91.4%, I^2^ = 0.00%), and temozolomide, capecitabine, and 177Lutetium-DOTA0-Tyr3-octreotate (177Lu-DOTATATE) with 75.1% (95% CI of 61.0% to 85.3%, I^2^ = 0.00%) and 98.0% (95% CI of 87.1% to 99.7%, I^2^ = 0.00%).

We assessed the potential of having publication bias in this systematic review and meta-analysis using Egger’s test. The Egger’s test showed that there were no significant potential publication biases for the estimation of any of the outcome measures; the p-values of this test for the pooled rate of CR, PR, SD, PD, ORR, and DCR were 0.17, 0.65, 0.86, 0.29, 0.98, 0.15, and 0.87, respectively. Furthermore, the results of the sensitivity analyses are presented in Supplementary Fig. 1, which revealed no significant alterations in the pooled outcomes.

### Quantitative analyses on the safety of temozolomide combination therapy

**Fig. 4:**
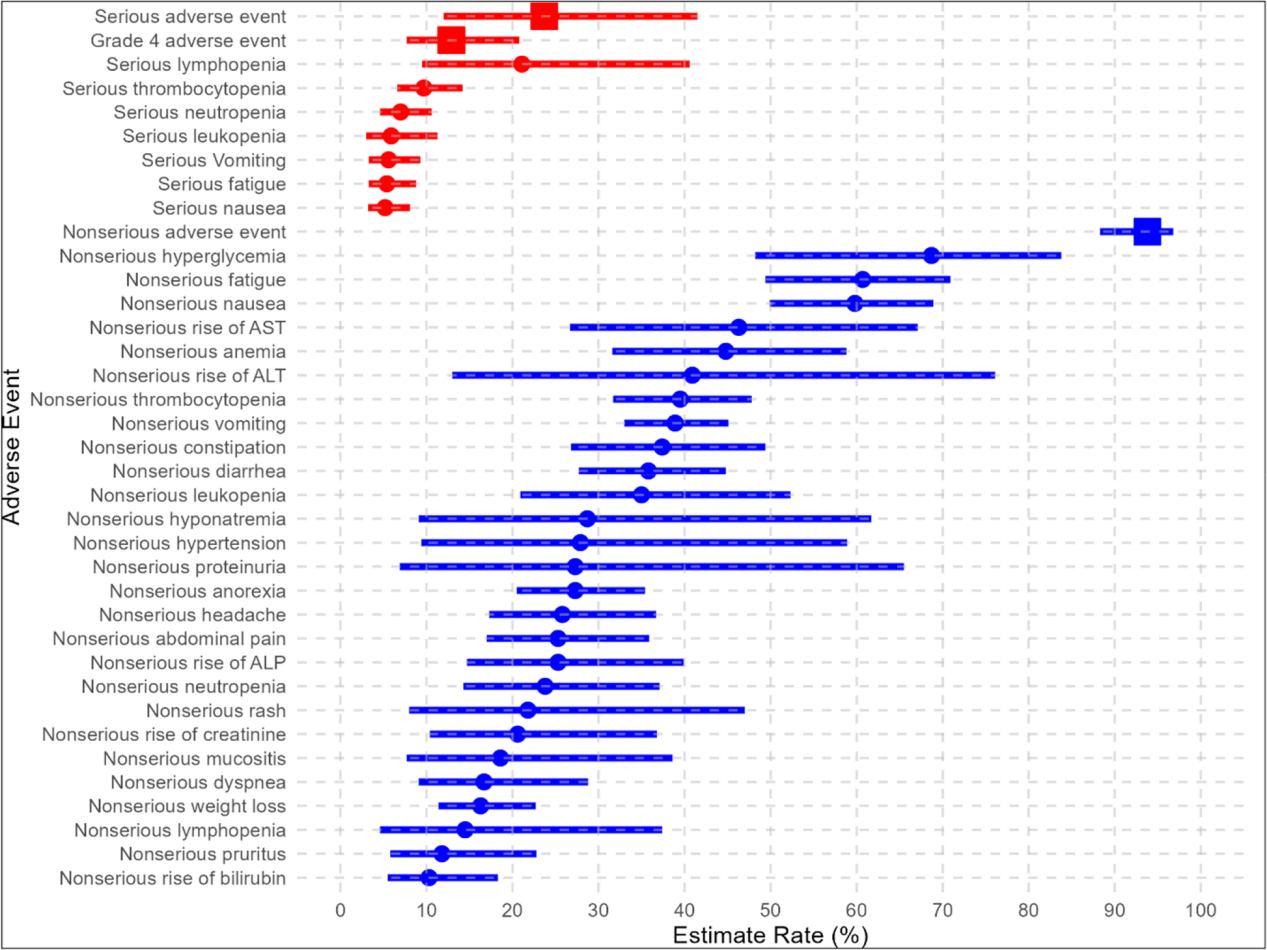
Serious and nonserious AEs observed in patients who received temozolomide-based treatment

The pooled rate of having at least one nonserious AE was 93.8% (95% CI of 88.3% to 96.8%, I^2^ = 15.8%) while for serious AEs, the rate was 23.7% (95% CI of 12.0% to 41.5%, I^2^ = 90.0%). The pooled rate of grade 4 AE was 12.9% (95% CI of 7.7% to 20.8%, I^2^ = 0.00%). Only in one of the included studies, there was a report of a patient with treatment-related grade 5 AE [31]. Figure 4 illustrates the individual pooled rates for each AE, along with their respective 95% CIs. The pooled rates, their 95% CI, and the I^2^ statistics used for estimating each pooled rate can be found in the Supplementary material. The main serious AEs in these patients who were on temozolomide-based treatment were hematologic AEs, including lymphopenia, 21.1%, thrombocytopenia, 9.7%, neutropenia, 7.0%, and leukopenia, 5.9%.

### Quality assessment

The comprehensive details regarding the quality ratings of the included studies can be found in Supplementary Tables 2 and 3. Notably, the overall assessment revealed that half of the included studies received a rating of ‘fair’, while four studies were rated as having ‘poor’ quality. None of the studies had blinding which was an item considered in the assessment tools. However, nearly all of the included studies consistently had explicitly defined objectives, well-defined inclusion and exclusion criteria, pre-specified outcome measures, and a low loss to follow-up rate, which did not exceed 20%.

## Discussion

The results of our systematic review and meta-analysis, based on data from 14 clinical trials, provide valuable insights into the efficacy and safety of temozolomide-based treatments for patients with advanced pNET. In this study, we assessed the efficacy with both radiographic and biochemical outcome measures. Our analysis revealed a pooled ORR of 41.2% (95% CI of 32.4% to 50.6%), indicating a substantial proportion of patients experiencing objective responses, either complete or partial, to treatment. The pooled DCR of 85.3% (95% CI of 74.9% to 91.9%) further underscores the potential benefits of these regimens in controlling the disease progression. Additionally, we found a pooled biochemical response of 44.9% (95% CI of 31.6% to 49.0%) which indicates a positive impact on reducing the chromogranin A levels, which is a sensitive and practical tumor biomarker commonly used for both the diagnosis and assessment of the response to the treatment in patients with pNET. Therefore, the results showed that temozolomide-based treatments could bring in promising outcomes for patients with advanced pNET. However, it's essential to acknowledge the presence of some nonserious and serious AEs, which require careful consideration when balancing the benefits and risks of these regimens in clinical practice. Moreover, given that the main serious AEs associated with temozolomide were hematological toxicities, close monitoring of blood counts should be integral to the clinical implementation of these treatment regimens.

The combination of temozolomide and capecitabine, as well as temozolomide and bevacizumab, although, demonstrated comparable effectiveness to temozolomide alone in terms of ORR, both combination therapies exhibited far higher DCR compared to temozolomide alone. This suggests that the combination therapies may be more effective than monotherapy. Patients who received temozolomide and capecitabine showed similar ORR and DCR compared to those on temozolomide and bevacizumab. Consequently, determining the optimal chemotherapy combination between temozolomide and capecitabine versus temozolomide and bevacizumab requires further investigation through additional studies. However, among all the treatments administered to the patients with advanced pNET in the included studies, the top two highest rates of ORR, 68%, and 80%, belonged to the Pavlakis et al. study [34] and Claringbold et al. study [28], respectively. In these two studies, the participants were on a combination of temozolomide, capecitabine, and 177Lu-DOTATATE. Moreover, the DCR in both these patients' groups was 100% indicating a promising efficacy of the combination of chemotherapy and radionuclide therapy. Both of these options, chemotherapy with temozolomide and PRRT with 177Lu-DOTATATE, could act independently to induce apoptosis by breaking the DNA structure. However, the combination of chemotherapy and radionuclides might result in additive to near-synergistic effects on tumoral cells, enhancing the efficacy while not increasing the toxicity as the chemotherapeutic agents could also exert radiosensitizing properties [38]. This improved efficacy is consistent with the findings observed on ORR and DCR among the included studies of this systematic review and meta-analysis. Studies have shown that agents such as temozolomide and 5-fluorouracil and its prodrug capecitabine can radiosensitize tumors to targeted radionuclide therapy and increase their cytotoxic effects [39–42]; besides, molecular studies also have demonstrated that there is an upregulation of somatostatin receptors type 2 and thereby an increased rate of tumoral uptake of somatostatin analogs with these agents [43, 44].

There are also three ongoing phase II studies on the effectiveness of a standard dose of 177Lu-DOTATATE and temozolomide-based chemotherapy. Two of these currently-recruiting studies are RCTs; one is solely in the United States on patients with well-differentiated pNETs (NCT05247905) [45] and the other is an international multicenter study on those with gastroenteropancreatic neuroendocrine tumors (NCT04919226) [46]; in both studies, the efficacy and safety of 177Lu-DOTATATE are being compared to chemotherapeutic regimens containing temozolomide. The third study is a Polish single arm study (NCT04194125) that has also been designed to assess the usefulness of 177Lu-DOTATATE in combination with temozolomide and capecitabine in patients with gastroenteropancreatic neuroendocrine tumors [47].

The effectiveness of temozolomide in the treatment of patients with pNET appears to be influenced by the O-6-methylguanine-DNA methyltransferase (MGMT) status. MGMT is a DNA repair enzyme that functions against the DNA methylation induced by the alkylating agents, such as temozolomide. The predictive value of MGMT deficiency in both prognosis and response to temozolomide in glioblastoma is well-established, which mainly occurs through the methylation of the promoter in patients with this tumor [48, 49]. The existing literature suggests that MGMT status may also be predictive of the response to temozolomide in patients with pNET. While retrospective studies on the significance of MGMT as a biochemical marker in pNET management presented inconsistent findings, recent evidence from two prospective RCTs on patients with advanced pNET provided robust evidence supporting the usefulness of MGMT status [50–53]. In this regard, Chi et al. demonstrated significantly higher ORR and extended PFS in individuals with negative MGMT status compared to those with positive status [36]. Similarly, Kunz et al. reported a heightened response rate to temozolomide in patients with negative MGMT status [37]. These findings emphasize the potential clinical relevance of assessing MGMT status in guiding temozolomide treatment decisions for pNET patients. Besides, a recent phase II RCT on patients with advanced NET indicated that alkylating agent-based chemotherapy was more effective in MGMT-deficient participants in terms of the best ORR, PFS, and OS [54]. Nevertheless, the available evidence remains inadequate to advocate for the routine testing of MGMT status in all patients with advanced pNET.

There are also several other ongoing phase II trials aiming to explore the use of temozolomide in combination with other anticancer medications for advanced neuroendocrine tumors. Two RCTs are targeting only individuals with advanced unresectable or metastatic gastroenteropancreatic neuroendocrine tumors. One trial in the United States (NCT02595424) compares temozolomide plus capecitabine with etoposide plus cisplatin or carboplatin [55], while another in China (NCT03279601) is assigning the patients to receive either dacarbazine plus capecitabine or temozolomide plus capecitabine [56]. The French BITTER 2 study (NCT03351296) is also an ongoing trial, assessing the efficacy and safety of two common chemotherapy regimens, temozolomide plus capecitabine and 5-fluorouracil plus streptozocin, both with or without bevacizumab [57]. These studies represent valuable efforts ongoing around the world to enhance the understanding of different temozolomide-based chemotherapy regimens in diverse patient populations.

Although there is a well-established body of level 2 or 3 evidence supporting the use of systemic medical treatment in patients with advanced unresectable pNET, a significant gap regarding the role of these therapies as adjuvant or neoadjuvant options exists for individuals with localized tumors. Moreover, studies conducted so far are predominantly retrospective and lack critical information about the specific chemotherapy regimens employed and other factors relevant and important to the clinical decision-making for these patients [58]. Consequently, these therapies have not yet been recommended in the current clinical practice guidelines [59, 60]. However, a phase II RCT (NCT05040360) has been initiated to evaluate the efficacy and safety of temozolomide as adjuvant chemotherapy [61, 62]. This study aims to assign well-differentiated pNET patients who have undergone surgical resection of the primary lesion to either receive temozolomide and capecitabine or not. Patients with a Zaidi score of 0 to 2, indicating a low risk of recurrence, are not included since the primary objective of the study is to assess recurrence-free survival [63]. This trial represents a critical step towards determining the potential role of temozolomide in the adjuvant setting for resectable localized pNET with a high risk for recurrence.

We must acknowledge some limitations in the current systematic review and meta-analysis. While a considerable number of studies were included in the quantitative analyses, the total number of included patients was relatively low; besides, most of the studies were single arm clinical trials. This issue restricted us from making comparisons regarding the efficacy and safety of different therapeutic regimens and estimating and pooling critical parameters such as hazard ratios. Therefore, we only were able to yield pooled proportions of the outcome measures for the efficacy evaluation. Besides, there was insufficient data in the included studies to perform analysis on two crucial outcome measures: PFS and overall survival. Moreover, there was a notable diversity in the combination of temozolomide-based treatment regimens across the studies which introduced substantial heterogeneity into the analyses. These limitations may affect the generalizability of our findings and highlight the need for larger well-designed studies in the future. There were also limitations in the evaluation of the safety profile of temozolomide. One significant limitation was that in most of the studies, temozolomide was given to the patients in combination with other treatment options. This complicates the attribution of AEs, especially nonserious and nonspecific ones, definitively to temozolomide, as they could potentially stem from the concurrent treatments administered to the patients. The other limitation in the safety profile evaluation pertains to the generalization of the pooled AE rates obtained through the quantitative analyses in this study. These rates may not accurately represent the real-world prevalence of AEs associated with temozolomide use, as they are derived exclusively from the data within the studies on patients with advanced NET, thereby constraining their broader applicability to the wider population of all patients who are receiving temozolomide treatment.

## Conclusions

In conclusion, our meta-analysis indicates that treatment with temozolomide, whether used alone or in combination with other anticancer therapies, could be an effective choice for patients with advanced pNET. Additionally, despite a relatively high rate of AEs associated with temozolomide-based treatment, the majority of these events were nonspecific and nonserious and the demonstrated rate of serious AEs suggests that the medication has an acceptable level of safety. These findings hold particular significance, especially considering the limited treatment options currently available for patients with advanced, locally unresectable, and metastatic pNET. To enhance the robustness of these findings, further clinical trials, particularly RCTs, are, however, essential. These trials should aim to compare the effectiveness and safety of various temozolomide-based regimens. Moreover, there is a need for studies that evaluate the efficacy of temozolomide-based regimens versus non-temozolomide-based regimens in patients with pNET.

### Supplementary Information

**Additional file 1.** Search strategy. **Supplementary Figure 1.** Sensitivity analyses for pooled effect sizes of (**a**) ORR, (**b**) DCR, and (**c**) having more than 50% decrease in chromogranin A levels. **Supplementary Table 2.** Quality assessment of the included single arm trials using NHLBI assessment. **Supplementary Table 3.** Quality assessment of the included controlled trials using NHLBI assessment.

## Data Availability

No datasets were generated or analysed during the current study.

## Abbreviations

AE: Adverse event
CI: Confidence interval
CR: Complete response
DCR: Disease control rate
DNA: Deoxyribonucleic acid
ORR: Objective response rate
PD: Progressive disease
PFS: Progression-free survival
pNET: Pancreatic neuroendocrine tumor
PR: Partial response
PRISMA: Preferred Reporting Items for Systematic Review and Meta-Analysis
PRRT: Peptide receptor radionuclide therapy
RCT: Randomized clinical trial
SD: Stable disease
