# Supplementary material for: Efficacy and safety of temozolomide-based regimens in advanced pancreatic neuroendocrine tumors: a systematic review and meta-analysis
Source: BMC Cancer. 2024 Feb 12;24:192. doi: 10.1186/s12885-024-11926-2 (PMC10860315; doi:10.1186/s12885-024-11926-2)
Supplement: Supplementary file 1 — Additional file 1. Search strategy. Supplementary Figure 1. Sensitivity analyses for pooled effect sizes of (a) ORR, (b) DCR, and (c) having more than 50% decrease in chromogranin A levels. Supplementary Table 2. Quality assessment of the included single arm trials using NHLBI assessment. Supplementary Table 3. Quality assessment of the included controlled trials using NHLBI assessment. [file 12885_2024_11926_MOESM1_ESM.docx]

**Supplementary material**

**Search strategy**----------------------------------------------------------------------------------------------------------2

**Supplementary Figure 1**-----------------------------------------------------------------------------------------------3

**Supplementary Table 1**------------------------------------------------------------------------------------------------4

**Supplementary Table 2**------------------------------------------------------------------------------------------------5

**Supplementary Table 3**------------------------------------------------------------------------------------------------7

**Search strategy**

**PubMed:**

(((((((((((Pancreatic neuroendocrine tumor[Title/Abstract]) OR (PNET[Title/Abstract])) OR (Pancreatic NET[Title/Abstract])) OR (islet cell carcinoma[Title/Abstract])) OR (islet cell tumor[Title/Abstract])) OR (Gastrinoma[Title/Abstract])) OR (Insulinoma[Title/Abstract])) OR (Glucagonoma[Title/Abstract])) OR (VIPoma[Title/Abstract])) OR (Somatostatinoma[Title/Abstract])) OR (pancreatic neoplasm[MeSH Terms])) AND (((((((((Temozolomide[Title/Abstract]) OR (Temodar[Title/Abstract])) OR (TMZ[Title/Abstract])) OR (Methazolastone[Title/Abstract])) OR (Temodal[Title/Abstract])) OR (CCRG 81045[Title/Abstract])) OR (NSC 362856[Title/Abstract])) OR (M and B 39831[Title/Abstract])) OR (M & B 39831[Title/Abstract])) Filters: Clinical Study, Clinical Trial, Clinical Trial, Phase I, Clinical Trial, Phase II, Clinical Trial, Phase III, Clinical Trial, Phase IV, Controlled Clinical Trial, Multicenter Study, Randomized Controlled Trial, English

**Embase:**

(('pancreatic neuroendocrine tumor':ti,ab,kw OR 'pancreatic neoplasm':ti,ab,kw OR pnet:ti,ab,kw OR 'pancreatic net':ti,ab,kw OR 'islet cell carcinoma':ti,ab,kw OR 'islet cell tumor':ti,ab,kw OR gastrinoma:ti,ab,kw OR insulinoma:ti,ab,kw OR glucagonoma:ti,ab,kw OR vipoma:ti,ab,kw OR somatostatinoma:ti,ab,kw) AND (temozolomide:ti,ab,kw OR temodar:ti,ab,kw OR tmz:ti,ab,kw OR methazolastone:ti,ab,kw OR temodal:ti,ab,kw OR 'ccrg 81045':ti,ab,kw OR 'nsc 362856':ti,ab,kw OR 'm and b 39831':ti,ab,kw OR 'm & b 39831':ti,ab,kw) AND ('clinical article'/de OR 'clinical study'/de OR 'clinical trial'/de OR 'clinical trial topic'/de OR 'comparative effectiveness'/de OR 'controlled clinical trial'/de OR 'controlled study'/de OR 'major clinical study'/de OR 'multicenter study'/de OR 'phase 1 clinical trial'/de OR 'phase 2 clinical trial'/de OR 'phase 2 clinical trial topic'/de OR 'phase 3 clinical trial'/de OR 'phase 3 clinical trial topic'/de OR 'pilot study'/de OR 'randomized controlled trial'/de OR 'randomized controlled trial topic'/de) AND ('article'/it OR 'article in press'/it) AND [english]/lim

**Web of Science:**

(Pancreatic neuroendocrine tumor (Topic) or Pancreatic neoplasm (Topic) or PNET (Topic) or Pancreatic NET (Topic) or islet cell carcinoma (Topic) or islet cell tumor (Topic) or Gastrinoma (Topic) or Insulinoma (Topic) or Glucagonoma (Topic) or VIPoma (Topic) or Somatostatinoma (Topic)) AND (Temozolomide (Topic) or Temodar (Topic) or TMZ (Topic) or Methazolastone (Topic) or Temodal (Topic) or CCRG 81045 (Topic) or NSC 362856 (Topic) or M and B 39831 (Topic) or M & B 39831 (Topic)) and Article (Document Types) and English (Languages)

**The Cochrane Library:**

#1 ((Pancreatic neuroendocrine tumor):ti,ab,kw OR (Pancreatic neoplasm):ti,ab,kw OR (PNET):ti,ab,kw OR (Pancreatic NET):ti,ab,kw OR (islet cell tumor):ti,ab,kw)

#2 MeSH descriptor: [Pancreatic Neoplasms] explode all trees

#3 (Temozolomide):ti,ab,kw OR (Temodar):ti,ab,kw OR (TMZ):ti,ab,kw OR (Methazolastone):ti,ab,kw OR (Temodal):ti,ab,kw

#4 MeSH descriptor: [Temozolomide] explode all trees

(#1 OR #2) AND (#3 OR #4)

******Supplementary Figure 1.** Sensitivity analyses for pooled effect sizes of **(a)** ORR, **(b)** DCR, and **(c)** having more than 50% decrease in chromogranin A levels

| **Supplementary Table 1.** Pooled effect sizes of serious and nonserious AEs observed in patients who received temozolomide-based treatment | | | | |
| --- | --- | --- | --- | --- |
| Adverse event | Estimate rate | Lower confidence interval | Upper confidence interval | Heterogeneity |
| Serious adverse event | 23.7% | 12.0% | 41.5% | 90.0% |
| Grade 4 adverse event | 12.9% | 7.7% | 20.8% | 0.0% |
| Serious lymphopenia | 21.1% | 9.5% | 40.6% | 89.0% |
| Serious thrombocytopenia | 9.7% | 6.6% | 14.2% | 36.7% |
| Serious neutropenia | 7.0% | 4.6% | 10.6% | 4.4% |
| Serious leukopenia | 5.9% | 3.0% | 11.3% | 48.7% |
| Serious Vomiting | 5.6% | 3.3% | 9.3% | 14.5% |
| Serious fatigue | 5.4% | 3.3% | 8.8% | 0.0% |
| Serious nausea | 5.2% | 3.2% | 8.1% | 0.0% |
| Nonserious adverse event | 93.8% | 88.3% | 96.8% | 15.8% |
| Nonserious hyperglycemia | 68.7% | 48.2% | 83.8% | 68.8% |
| Nonserious fatigue | 60.7% | 49.4% | 70.9% | 74.5% |
| Nonserious nausea | 59.8% | 49.9% | 68.9% | 66.8% |
| Nonserious rise of AST | 46.3% | 26.7% | 67.1% | 82.8% |
| Nonserious anemia | 44.8% | 31.6% | 58.8% | 82.0% |
| Nonserious rise of ALT | 40.9% | 13.0% | 76.1% | 91.2% |
| Nonserious thrombocytopenia | 39.5% | 31.7% | 47.8% | 56.1% |
| Nonserious vomiting | 38.9% | 33.0% | 45.1% | 26.0% |
| Nonserious constipation | 37.4% | 26.8% | 49.4% | 73.5% |
| Nonserious diarrhea | 35.8% | 27.7% | 44.8% | 60.7% |
| Nonserious leukopenia | 35.0% | 20.9% | 52.3% | 80.6% |
| Nonserious hyponatremia | 28.7% | 9.1% | 61.7% | 87.7% |
| Nonserious hypertension | 27.9% | 9.4% | 58.9% | 88.2% |
| Nonserious proteinuria | 27.3% | 6.9% | 65.5% | 88.0% |
| Nonserious anorexia | 27.3% | 20.5% | 35.4% | 48.8% |
| Nonserious headache | 25.8% | 17.3% | 36.7% | 71.8% |
| Nonserious abdominal pain | 25.3% | 17.0% | 35.9% | 72.4% |
| Nonserious rise of ALP | 25.3% | 14.7% | 39.9% | 71.0% |
| Nonserious neutropenia | 23.8% | 14.3% | 37.1% | 81.2% |
| Nonserious rash | 21.8% | 8.0% | 47.0% | 87.2% |
| Nonserious rise of creatinine | 20.6% | 10.4% | 36.8% | 74.9% |
| Nonserious mucositis | 18.6% | 7.7% | 38.6% | 88.2% |
| Nonserious dyspnea | 16.7% | 9.1% | 28.8% | 52.4% |
| Nonserious weight loss | 16.3% | 11.4% | 22.7% | 0.0% |
| Nonserious lymphopenia | 14.5% | 4.6% | 37.4% | 90.1% |
| Nonserious pruritus | 11.8% | 5.8% | 22.8% | 66.2% |
| Nonserious rise of bilirubin | 10.3% | 5.5% | 18.3% | 58.4% |

| **Supplementary Table 2.** Quality assessment of the included single arm trials using NHLBI assessment | | | | | | | | | | |
| --- | --- | --- | --- | --- | --- | --- | --- | --- | --- | --- |
| Items | Kulke et al. (a) | Kulke et al. (b) | Chan et al. (a) 2012 | Chan et al. (b) 2013 | Fine et al. | Claringbold et al. | Pavel et al., | Bhave et al. | Shaheen et al. | Kobayashi et al. |
| 1. Was the study question or objective clearly stated? | Y | Y | Y | Y | Y | Y | Y | Y | Y | Y |
| 2. Were eligibility/selection criteria for the study population prespecified and clearly described? | Y | CD | Y | Y | Y | Y | Y | Y | Y | Y |
| 3. Were the participants in the study representative of those who would be eligible for the test/service/intervention in the general or clinical population of interest? | Y | CD | Y | Y | Y | Y | Y | Y | Y | Y |
| 4. Were all eligible participants that met the prespecified entry criteria enrolled? | Y | CD | Y | Y | Y | Y | Y | Y | Y | Y |
| 5. Was the sample size sufficiently large to provide confidence in the findings? | Y | CD | Y | Y | Y | CD | Y | N | N | N |
| 6. Was the test/service/intervention clearly described and delivered consistently across the study population? | Y | CD | Y | Y | Y | Y | Y | Y | Y | Y |
| 7. Were the outcome measures prespecified, clearly defined, valid, reliable, and assessed consistently across all study participants? | Y | Y | Y | Y | Y | Y | Y | Y | Y | Y |
| 8. Were the people assessing the outcomes blinded to the participants' exposures/interventions? | N | N | N | N | N | N | N | N | N | N |
| 9. Was the loss to follow-up after baseline 20% or less? Were those lost to follow-up accounted for in the analysis? | Y, NA | Y, N | Y, NA | Y, N | N, N | Y, NA | N, Y | Y, N | Y, N | Y, NA |
| 10. Did the statistical methods examine changes in outcome measures from before to after the intervention? Were statistical tests done that provided p values for the pre-to-post changes? | N | N | N | N | N | N | N | N | N | Y |
| 11. Were outcome measures of interest taken multiple times before the intervention and multiple times after the intervention (i.e., did they use an interrupted time-series design)? | Y | Y | Y | Y | Y | Y | Y | Y | Y | Y |
| 12. If the intervention was conducted at a group level (e.g., a whole hospital, a community, etc.) did the statistical analysis take into account the use of individual-level data to determine effects at the group level? | NA | NA | NA | NA | NA | NA | NA | NA | NA | NA |
| Overall quality | Fair | Poor | Good | Fair | Fair | Good | Fair | Poor | Fair | Fair |

Abbreviations: CD, cannot determine; N, no; NA, not applicable; Y, yes.

| **Supplementary Table 3.** Quality assessment of the included controlled trials using NHLBI assessment | | | | |
| --- | --- | --- | --- | --- |
| Items | Chi et al. | Kunz et al. | Pavlakis et al. | Cheng et al. |
| 1. Was the study described as randomized, a randomized trial, a randomized clinical trial, or an RCT? | Y | Y | Y | N |
| 2. Was the method of randomization adequate (i.e., use of randomly generated assignment)? | Y | NR | NR | NA |
| 3. Was the treatment allocation concealed (so that assignments could not be predicted)? | N | NR | NR | N |
| 4. Were study participants and providers blinded to treatment group assignment? | N | N | N | N |
| 5. Were the people assessing the outcomes blinded to the participants' group assignments? | N | N | N | N |
| 6. Were the groups similar at baseline on important characteristics that could affect outcomes (e.g., demographics, risk factors, co-morbid conditions)? | Y | Y | NR | NR |
| 7. Was the overall drop-out rate from the study at endpoint 20% or lower of the number allocated to treatment? | Y | Y | Y | Y |
| 8. Was the differential drop-out rate (between treatment groups) at endpoint 15 percentage points or lower? | Y | Y | Y | Y |
| 9. Was there high adherence to the intervention protocols for each treatment group? | Y | Y | NR | Y |
| 10. Were other interventions avoided or similar in the groups (e.g., similar background treatments)? | Y | Y | Y | Y |
| 11. Were outcomes assessed using valid and reliable measures, implemented consistently across all study participants? | Y | Y | Y | Y |
| 12. Did the authors report that the sample size was sufficiently large to be able to detect a difference in the main outcome between groups with at least 80% power? | Y | Y | N | Y |
| 13. Were outcomes reported or subgroups analyzed prespecified (i.e., identified before analyses were conducted)? | Y | Y | Y | Y |
| 14. Were all randomized participants analyzed in the group to which they were originally assigned, i.e., did they use an intention-to-treat analysis? | Y | N | N | N |
| Overall quality | Good | Fair | Poor | Poor |

Abbreviations: N, no; NA, not applicable; NR, not reported; Y, yes.
